# Supplementary material for: Endosymbiont interference and microbial diversity of the Pacific coast tick, Dermacentor occidentalis, in San Diego County, California
Source: PeerJ. 2017 Apr 13;5:e3202. doi: 10.7717/peerj.3202 (PMC5426561; doi:10.7717/peerj.3202)
Supplement: Table S1 — Spotted Fever group Rickettsia identified by rompA and IGR sequencing and total number of individual 16S rRNA gene sequences from each tick. [file peerj-05-3202-s001.docx]

Supplemental table 1. Spotted Fever group *Rickettsia* identified and total number of individual 16S rRNA gene sequences from each tick.

| Tick | Location | SFGR ID | No. sequences |
| --- | --- | --- | --- |
| T14.0091 | Mission Trails |  | 48409 |
| T14.0092 | Mission Trails | R.rhipicephali | 55867 |
| T14.0093 | Mission Trails |  | 24444 |
| T14.0094 | Mission Trails |  | 66297 |
| T14.0095 | Mission Trails |  | 87380 |
| T14.0096 | Mission Trails |  | 10803 |
| T14.0101 | Mission Trails |  | 58369 |
| T14.0102 | Mission Trails |  | 13288 |
| T14.0103 | Mission Trails |  | 65361 |
| T14.0106 | Mission Trails |  | 41139 |
| T14.0107 | Mission Trails |  | 38876 |
| T14.0215 | Lopez Canyon |  | 3699 |
| T14.0216 | Lopez Canyon |  | 68183 |
| T14.0218 | Lopez Canyon |  | 61577 |
| T14.0219 | Lopez Canyon |  | 74014 |
| T14.0224 | Lopez Canyon |  | 88047 |
| T14.0225 | Lopez Canyon |  | 4767 |
| T14.0226 | Lopez Canyon |  | 49576 |
| T14.0227 | Lopez Canyon |  | 47685 |
| T14.0228 | Lopez Canyon |  | 36930 |
| T14.0268 | Lopez Canyon | R.philipii | 78687 |
| T14.0274 | Lopez Canyon | R. rhipicephali | 119650 |
| T14.0278 | Lopez Canyon | R.rhipicephali | 96916 |
| T14.0283 | Lopez Canyon | R. rhipicephali | 20188 |
| T14.0288 | Lopez Canyon | R. rhipicephali | 68237 |
| T14.0302 | Lopez Canyon | R. rhipicephali | 32371 |
| T14.0311 | Lopez Canyon | R.rhipicephali | 142563 |
| T14.0323 | Escondido Creek | R.rhipicephali | 41069 |
| T14.0327 | Escondido Creek | R.rhipicephali | 96110 |
| T14.0329 | Escondido Creek | R.philipii | 66060 |
| T14.0330 | Escondido Creek |  | 73657 |
| T14.0331 | Escondido Creek |  | 46351 |
| T14.0334 | Escondido Creek |  | 12990 |
| T14.0335 | Escondido Creek |  | 42845 |
| T14.0340 | Escondido Creek | R.rhipicephali | 30688 |
| T14.0350 | Escondido Creek | R.rhipicephali | 58470 |
| T14.0365 | Escondido Creek | R.rhipicephali | 97560 |
| T14.0366 | Escondido Creek |  | 88178 |
| T14.0367 | Escondido Creek |  | 82780 |
| T14.0368 | Escondido Creek |  | 58058 |
| T14.0369 | Escondido Creek |  | 57198 |
| T14.0370 | Escondido Creek |  | 81748 |
| T14.0381 | Escondido Creek | R.rhipicephali | 50800 |
| T14.0388 | Escondido Creek | R.rhipicephali | 82511 |
| T14.0399 | Penasquitos Canyon | R.rhipicephali | 98911 |
| T14.0404 | Penasquitos Canyon | R.rhipicephali | 36606 |
| T14.0407 | Penasquitos Canyon | R.rhipicephali | 8526 |
| T14.0408 | Penasquitos Canyon |  | 68734 |
| T14.0409 | Penasquitos Canyon |  | 43102 |
| T14.0410 | Penasquitos Canyon |  | 113854 |
| T14.0411 | Penasquitos Canyon |  | 66105 |
| T14.0418 | Penasquitos Canyon | R.rhipicephali | 56439 |
| T14.0419 | Penasquitos Canyon | R.rhipicephali | 125331 |
| T14.0420 | Penasquitos Canyon |  | 61818 |
| T14.0422 | Penasquitos Canyon |  | 137433 |
| T14.0423 | Penasquitos Canyon |  | 82919 |
| T14.0428 | Penasquitos Canyon | R.rhipicephali | 29414 |
| T14.0433 | Penasquitos Canyon | R.rhipicephali | 128714 |
| T14.0434 | Penasquitos Canyon |  | 88454 |
| T14.0436 | Penasquitos Canyon |  | 60751 |
| T14.0592 | Lopez Canyon |  | 68864 |
| T14.0602 | Lopez Canyon | R.philipii | 102889 |
| T14.0603 | Lopez Canyon | R.rhipicephali | 108402 |
| T14.0625 | Lopez Canyon | R.philipii | 43244 |
| T14.0626 | Lopez Canyon | R.philipii | 97349 |
| T14.0637 | Lopez Canyon | R.philipii | 62484 |
| T14.0639 | Lopez Canyon | R.philipii | 93587 |
| T14.0640 | Lopez Canyon |  | 138880 |
| T14.0641 | Lopez Canyon |  | 42241 |
| T14.0642 | Lopez Canyon |  | 91657 |
| T14.0644 | Lopez Canyon | R.rhipicephali | 35278 |
| T14.0645 | Lopez Canyon |  | 95138 |
| T14.0646 | Lopez Canyon |  | 250403 |
| T14.0647 | Lopez Canyon |  | 67944 |
| T14.0648 | Lopez Canyon |  | 43399 |
| T14.0651 | Lopez Canyon | R.rhipicephali | 79939 |
| T14.0652 | Lopez Canyon | R.philipii | 102212 |
| T14.0657 | Lopez Canyon | R.philipii | 24374 |
| T14.0660 | Lopez Canyon | R.philipii | 22449 |
| T14.0665 | Lopez Canyon | R.rhipicephali | 57326 |
| T14.0666 | Lopez Canyon |  | 87494 |
| T14.0667 | Lopez Canyon |  | 105711 |
| T14.0669 | Lopez Canyon |  | 106398 |
| T14.0670 | Lopez Canyon |  | 58988 |
| T14.0682 | Lopez Canyon | R.rhipicephali | 34666 |
| T14.0696 | Lopez Canyon | R.rhipicephali | 97800 |
| T14.0700 | Lopez Canyon | R.rhipicephali | 106720 |
| T14.0710 | Lopez Canyon | R.rhipicephali | 106684 |
| T14.0717 | Lopez Canyon | R.philipii | 69047 |
| T14.0730 | Mission Trails | R. rhipicephali | 47750 |
| T14.0762 | Mission Trails | R. rhipicephali | 91329 |
| T14.0763 | Mission Trails |  | 14772 |
| T14.0764 | Mission Trails |  | 82606 |
| T14.0765 | Mission Trails |  | 2013 |
| T14.0766 | Mission Trails |  | 15661 |
| T14.0767 | Mission Trails |  | 77976 |
| T14.0768 | Mission Trails | R.rhipicephali | 43455 |
| T14.0769 | Mission Trails |  | 93720 |
| T14.0770 | Mission Trails |  | 37599 |
| T14.0773 | Mission Trails |  | 70691 |
| T14.0774 | Mission Trails |  | 28299 |
| T14.0775 | Mission Trails | R.philipii | 14981 |
