# Supplementary material for: Endosymbiont interference and microbial diversity of the Pacific coast tick, Dermacentor occidentalis, in San Diego County, California
Source: PeerJ. 2017 Apr 13;5:e3202. doi: 10.7717/peerj.3202 (PMC5426561; doi:10.7717/peerj.3202)
Supplement: Table S2 — EC, Escondido Creek; LC, Lopez Canyon; MT, Mission Trails, PC, Peñasquitos Canyon. [file peerj-05-3202-s002.docx]

Supplemental table 2. SourceTracker results for *D. occidentalis* microbiomes from San Diego County. EC=Escondido Creek; LC=Lopez Canyon; MT=Mission Trails, PC=Peñasquitos Canyon.

| Sample | SFG | Location | Control | Dog skin | Fish skin | Frog skin | Human skin | Iguana skin | Human oral | Pigeon skin | Plant | Rat skin | Soil | Unk |
| --- | --- | --- | --- | --- | --- | --- | --- | --- | --- | --- | --- | --- | --- | --- |
| T14.0330 | neg | EC | 0.0000 | 0.0000 | 0.0000 | 0.0000 | 0.0000 | 0.0000 | 0.0000 | 0.0000 | 0.0000 | 0.0000 | 0.0000 | 1.0000 |
| T14.0331 | neg | EC | 0.0000 | 0.0000 | 0.0000 | 0.0000 | 0.0000 | 0.0000 | 0.0000 | 0.0000 | 0.0000 | 0.0000 | 0.0000 | 1.0000 |
| T14.0334 | neg | EC | 0.0000 | 0.0610 | 0.0000 | 0.0000 | 0.0001 | 0.0000 | 0.0000 | 0.0000 | 0.0000 | 0.0001 | 0.0001 | 0.9387 |
| T14.0335 | neg | EC | 0.0000 | 0.0000 | 0.0000 | 0.0000 | 0.0000 | 0.0003 | 0.0000 | 0.0000 | 0.0000 | 0.0000 | 0.0000 | 0.9997 |
| T14.0366 | neg | EC | 0.0000 | 0.0001 | 0.0000 | 0.0000 | 0.0000 | 0.0000 | 0.0000 | 0.0000 | 0.0000 | 0.0000 | 0.0001 | 0.9998 |
| T14.0367 | neg | EC | 0.0000 | 0.0000 | 0.0001 | 0.0000 | 0.0000 | 0.0008 | 0.0000 | 0.0000 | 0.0001 | 0.0000 | 0.0000 | 0.9990 |
| T14.0368 | neg | EC | 0.0000 | 0.0323 | 0.0000 | 0.0000 | 0.0000 | 0.0043 | 0.0000 | 0.0000 | 0.0005 | 0.0000 | 0.0017 | 0.9612 |
| T14.0369 | neg | EC | 0.0000 | 0.0110 | 0.0000 | 0.0001 | 0.0001 | 0.0000 | 0.0000 | 0.0000 | 0.0000 | 0.0000 | 0.0000 | 0.9888 |
| T14.0370 | neg | EC | 0.0000 | 0.0000 | 0.0000 | 0.0000 | 0.0000 | 0.0000 | 0.0000 | 0.0000 | 0.0001 | 0.0000 | 0.0000 | 0.9999 |
| T14.0329 | R.philipii | EC | 0.0000 | 0.0000 | 0.0000 | 0.0000 | 0.0000 | 0.0000 | 0.0000 | 0.0000 | 0.0000 | 0.0000 | 0.0000 | 1.0000 |
| T14.0323 | R.rhipicephali | EC | 0.0000 | 0.0002 | 0.0000 | 0.0000 | 0.0001 | 0.0000 | 0.0000 | 0.0000 | 0.0000 | 0.0000 | 0.0000 | 0.9997 |
| T14.0327 | R.rhipicephali | EC | 0.0000 | 0.0000 | 0.0000 | 0.0000 | 0.0001 | 0.0000 | 0.0000 | 0.0000 | 0.0000 | 0.0000 | 0.0000 | 0.9999 |
| T14.0340 | R.rhipicephali | EC | 0.0000 | 0.0015 | 0.0000 | 0.0000 | 0.0000 | 0.0000 | 0.0000 | 0.0000 | 0.0002 | 0.0000 | 0.0000 | 0.9983 |
| T14.0350 | R.rhipicephali | EC | 0.0000 | 0.0000 | 0.0000 | 0.0000 | 0.0000 | 0.0000 | 0.0000 | 0.0000 | 0.0000 | 0.0000 | 0.0000 | 1.0000 |
| T14.0365 | R.rhipicephali | EC | 0.0000 | 0.0000 | 0.0000 | 0.0000 | 0.0000 | 0.0000 | 0.0000 | 0.0000 | 0.0000 | 0.0000 | 0.0000 | 1.0000 |
| T14.0381 | R.rhipicephali | EC | 0.0000 | 0.0000 | 0.0000 | 0.0000 | 0.0001 | 0.0000 | 0.0000 | 0.0000 | 0.0000 | 0.0000 | 0.0000 | 0.9999 |
| T14.0388 | R.rhipicephali | EC | 0.0000 | 0.0000 | 0.0000 | 0.0000 | 0.0000 | 0.0000 | 0.0000 | 0.0000 | 0.0000 | 0.0000 | 0.0000 | 1.0000 |
| T14.0215 | neg | LC | 0.0000 | 0.0000 | 0.0000 | 0.0000 | 0.0000 | 0.0000 | 0.0000 | 0.0000 | 0.0000 | 0.0000 | 0.0000 | 1.0000 |
| T14.0216 | neg | LC | 0.0000 | 0.0226 | 0.0001 | 0.0000 | 0.0001 | 0.0000 | 0.0000 | 0.0000 | 0.0000 | 0.0000 | 0.0003 | 0.9768 |
| T14.0218 | neg | LC | 0.0000 | 0.0326 | 0.0000 | 0.0000 | 0.0001 | 0.0000 | 0.0000 | 0.0003 | 0.0000 | 0.0000 | 0.0003 | 0.9667 |
| T14.0219 | neg | LC | 0.0000 | 0.2071 | 0.0001 | 0.0000 | 0.0000 | 0.0000 | 0.0000 | 0.0002 | 0.0000 | 0.0000 | 0.0005 | 0.7921 |
| T14.0224 | neg | LC | 0.0000 | 0.1967 | 0.0000 | 0.0000 | 0.0001 | 0.0001 | 0.0000 | 0.0000 | 0.0000 | 0.0000 | 0.0000 | 0.8031 |
| T14.0225 | neg | LC | 0.0000 | 0.0000 | 0.0000 | 0.0000 | 0.0000 | 0.0000 | 0.0000 | 0.0000 | 0.0000 | 0.0000 | 0.0000 | 1.0000 |
| T14.0226 | neg | LC | 0.0000 | 0.1256 | 0.0000 | 0.0000 | 0.0000 | 0.0000 | 0.0000 | 0.0000 | 0.0000 | 0.0000 | 0.0001 | 0.8743 |
| T14.0227 | neg | LC | 0.0000 | 0.0017 | 0.0000 | 0.0000 | 0.0001 | 0.0000 | 0.0000 | 0.0000 | 0.0000 | 0.0000 | 0.0000 | 0.9982 |
| T14.0228 | neg | LC | 0.0000 | 0.0253 | 0.0000 | 0.0000 | 0.0000 | 0.0000 | 0.0000 | 0.0000 | 0.0000 | 0.0000 | 0.0000 | 0.9747 |
| T14.0592 | neg | LC | 0.0000 | 0.0437 | 0.0000 | 0.0000 | 0.0000 | 0.0000 | 0.0000 | 0.0000 | 0.0000 | 0.0000 | 0.0002 | 0.9561 |
| T14.0640 | neg | LC | 0.0000 | 0.1252 | 0.0001 | 0.0000 | 0.0000 | 0.0003 | 0.0000 | 0.0000 | 0.0000 | 0.0000 | 0.0000 | 0.8744 |
| T14.0641 | neg | LC | 0.0000 | 0.1105 | 0.0000 | 0.0000 | 0.0000 | 0.0002 | 0.0000 | 0.0000 | 0.0001 | 0.0000 | 0.0000 | 0.8892 |
| T14.0642 | neg | LC | 0.0000 | 0.0698 | 0.0005 | 0.0002 | 0.0005 | 0.0001 | 0.0000 | 0.0000 | 0.0004 | 0.0002 | 0.0001 | 0.9281 |
| T14.0645 | neg | LC | 0.0000 | 0.1831 | 0.0000 | 0.0000 | 0.0003 | 0.0006 | 0.0000 | 0.0000 | 0.0002 | 0.0000 | 0.0000 | 0.8158 |
| T14.0646 | neg | LC | 0.0000 | 0.1442 | 0.0000 | 0.0000 | 0.0018 | 0.0006 | 0.0000 | 0.0000 | 0.0000 | 0.0000 | 0.0000 | 0.8534 |
| T14.0647 | neg | LC | 0.0000 | 0.1037 | 0.0000 | 0.0000 | 0.0000 | 0.0000 | 0.0000 | 0.0000 | 0.0000 | 0.0000 | 0.0000 | 0.8963 |
| T14.0648 | neg | LC | 0.0000 | 0.2927 | 0.0002 | 0.0000 | 0.0000 | 0.0048 | 0.0000 | 0.0000 | 0.0000 | 0.0000 | 0.0000 | 0.7023 |
| T14.0666 | neg | LC | 0.0000 | 0.0480 | 0.0000 | 0.0000 | 0.0000 | 0.0000 | 0.0000 | 0.0000 | 0.0000 | 0.0001 | 0.0001 | 0.9518 |
| T14.0667 | neg | LC | 0.0000 | 0.0000 | 0.0000 | 0.0000 | 0.0000 | 0.0000 | 0.0000 | 0.0000 | 0.0000 | 0.0000 | 0.0000 | 1.0000 |
| T14.0669 | neg | LC | 0.0000 | 0.0000 | 0.0000 | 0.0000 | 0.0000 | 0.0008 | 0.0000 | 0.0000 | 0.0000 | 0.0000 | 0.0000 | 0.9992 |
| T14.0670 | neg | LC | 0.0000 | 0.0021 | 0.0000 | 0.0000 | 0.0033 | 0.0003 | 0.0000 | 0.0000 | 0.0010 | 0.0000 | 0.0000 | 0.9933 |
| T14.0268 | R.philipii | LC | 0.0000 | 0.0021 | 0.0000 | 0.0000 | 0.0001 | 0.0002 | 0.0000 | 0.0000 | 0.0002 | 0.0000 | 0.0000 | 0.9974 |
| T14.0602 | R.philipii | LC | 0.0000 | 0.0005 | 0.0000 | 0.0000 | 0.0000 | 0.0000 | 0.0000 | 0.0000 | 0.0000 | 0.0000 | 0.0000 | 0.9995 |
| T14.0625 | R.philipii | LC | 0.0000 | 0.0000 | 0.0000 | 0.0000 | 0.0001 | 0.0000 | 0.0000 | 0.0000 | 0.0000 | 0.0000 | 0.0000 | 0.9999 |
| T14.0626 | R.philipii | LC | 0.0000 | 0.0001 | 0.0000 | 0.0000 | 0.0004 | 0.0000 | 0.0000 | 0.0001 | 0.0000 | 0.0000 | 0.0000 | 0.9994 |
| T14.0637 | R.philipii | LC | 0.0000 | 0.0001 | 0.0000 | 0.0000 | 0.0002 | 0.0000 | 0.0000 | 0.0000 | 0.0000 | 0.0000 | 0.0002 | 0.9995 |
| T14.0639 | R.philipii | LC | 0.0000 | 0.0000 | 0.0000 | 0.0000 | 0.0000 | 0.0000 | 0.0000 | 0.0000 | 0.0000 | 0.0000 | 0.0000 | 1.0000 |
| T14.0652 | R.philipii | LC | 0.0000 | 0.0000 | 0.0000 | 0.0000 | 0.0000 | 0.0000 | 0.0000 | 0.0000 | 0.0000 | 0.0000 | 0.0000 | 1.0000 |
| T14.0657 | R.philipii | LC | 0.0000 | 0.0000 | 0.0000 | 0.0000 | 0.0001 | 0.0000 | 0.0000 | 0.0000 | 0.0000 | 0.0000 | 0.0001 | 0.9998 |
| T14.0660 | R.philipii | LC | 0.0000 | 0.0000 | 0.0000 | 0.0000 | 0.0001 | 0.0000 | 0.0000 | 0.0000 | 0.0000 | 0.0000 | 0.0000 | 0.9999 |
| T14.0717 | R.philipii | LC | 0.0000 | 0.0000 | 0.0000 | 0.0000 | 0.0000 | 0.0000 | 0.0000 | 0.0000 | 0.0000 | 0.0000 | 0.0000 | 1.0000 |
| T14.0274 | R.rhipicephali | LC | 0.0000 | 0.1270 | 0.0000 | 0.0000 | 0.0119 | 0.0000 | 0.0000 | 0.0000 | 0.0000 | 0.0000 | 0.0000 | 0.8611 |
| T14.0278 | R.rhipicephali | LC | 0.0000 | 0.0562 | 0.0000 | 0.0000 | 0.0000 | 0.0000 | 0.0000 | 0.0000 | 0.0000 | 0.0000 | 0.0000 | 0.9438 |
| T14.0283 | R.rhipicephali | LC | 0.0000 | 0.1027 | 0.0001 | 0.0000 | 0.0000 | 0.0000 | 0.0000 | 0.0000 | 0.0003 | 0.0000 | 0.0008 | 0.8961 |
| T14.0288 | R.rhipicephali | LC | 0.0000 | 0.0627 | 0.0000 | 0.0000 | 0.0000 | 0.0000 | 0.0000 | 0.0000 | 0.0002 | 0.0000 | 0.0000 | 0.9371 |
| T14.0302 | R.rhipicephali | LC | 0.0000 | 0.0002 | 0.0000 | 0.0000 | 0.0000 | 0.0000 | 0.0000 | 0.0000 | 0.0000 | 0.0000 | 0.0000 | 0.9998 |
| T14.0311 | R.rhipicephali | LC | 0.0000 | 0.0753 | 0.0000 | 0.0000 | 0.0001 | 0.0000 | 0.0000 | 0.0000 | 0.0001 | 0.0000 | 0.0001 | 0.9244 |
| T14.0603 | R.rhipicephali | LC | 0.0000 | 0.0513 | 0.0000 | 0.0000 | 0.0000 | 0.0000 | 0.0000 | 0.0000 | 0.0000 | 0.0000 | 0.0001 | 0.9486 |
| T14.0644 | R.rhipicephali | LC | 0.0000 | 0.0000 | 0.0000 | 0.0000 | 0.0000 | 0.0000 | 0.0000 | 0.0000 | 0.0000 | 0.0000 | 0.0000 | 1.0000 |
| T14.0651 | R.rhipicephali | LC | 0.0000 | 0.0000 | 0.0000 | 0.0000 | 0.0000 | 0.0000 | 0.0000 | 0.0000 | 0.0000 | 0.0000 | 0.0000 | 1.0000 |
| T14.0665 | R.rhipicephali | LC | 0.0000 | 0.0000 | 0.0000 | 0.0000 | 0.0000 | 0.0000 | 0.0000 | 0.0000 | 0.0000 | 0.0000 | 0.0000 | 1.0000 |
| T14.0682 | R.rhipicephali | LC | 0.0000 | 0.0000 | 0.0000 | 0.0000 | 0.0000 | 0.0000 | 0.0000 | 0.0000 | 0.0000 | 0.0000 | 0.0000 | 1.0000 |
| T14.0696 | R.rhipicephali | LC | 0.0000 | 0.0836 | 0.0000 | 0.0000 | 0.0001 | 0.0000 | 0.0000 | 0.0000 | 0.0000 | 0.0000 | 0.0000 | 0.9163 |
| T14.0700 | R.rhipicephali | LC | 0.0000 | 0.0221 | 0.0000 | 0.0000 | 0.0001 | 0.0000 | 0.0000 | 0.0000 | 0.0001 | 0.0000 | 0.0000 | 0.9777 |
| T14.0710 | R.rhipicephali | LC | 0.0000 | 0.0000 | 0.0000 | 0.0000 | 0.0001 | 0.0000 | 0.0000 | 0.0000 | 0.0000 | 0.0000 | 0.0000 | 0.9999 |
| T14.0091 | neg | MT | 0.0000 | 0.2995 | 0.0000 | 0.0000 | 0.0000 | 0.0000 | 0.0000 | 0.0001 | 0.0000 | 0.0001 | 0.0006 | 0.6995 |
| T14.0093 | neg | MT | 0.0000 | 0.0000 | 0.0000 | 0.0000 | 0.0000 | 0.0000 | 0.0000 | 0.0000 | 0.0000 | 0.0000 | 0.0000 | 1.0000 |
| T14.0094 | neg | MT | 0.0000 | 0.0007 | 0.0001 | 0.0001 | 0.0000 | 0.0003 | 0.0000 | 0.0000 | 0.0000 | 0.0000 | 0.0000 | 0.9988 |
| T14.0095 | neg | MT | 0.0000 | 0.1547 | 0.0000 | 0.0000 | 0.0001 | 0.0001 | 0.0000 | 0.0000 | 0.0002 | 0.0005 | 0.0005 | 0.8439 |
| T14.0096 | neg | MT | 0.0000 | 0.0000 | 0.0000 | 0.0000 | 0.0000 | 0.0000 | 0.0000 | 0.0000 | 0.0000 | 0.0000 | 0.0001 | 0.9999 |
| T14.0101 | neg | MT | 0.0000 | 0.0000 | 0.0000 | 0.0000 | 0.0000 | 0.0000 | 0.0000 | 0.0000 | 0.0000 | 0.0000 | 0.0000 | 1.0000 |
| T14.0102 | neg | MT | 0.0000 | 0.0000 | 0.0000 | 0.0000 | 0.0003 | 0.0000 | 0.0000 | 0.0000 | 0.0001 | 0.0000 | 0.0000 | 0.9996 |
| T14.0103 | neg | MT | 0.0000 | 0.0000 | 0.0000 | 0.0000 | 0.0000 | 0.0000 | 0.0000 | 0.0000 | 0.0001 | 0.0000 | 0.0000 | 0.9999 |
| T14.0106 | neg | MT | 0.0000 | 0.0008 | 0.0000 | 0.0000 | 0.0000 | 0.0001 | 0.0000 | 0.0000 | 0.0000 | 0.0001 | 0.0000 | 0.9990 |
| T14.0107 | neg | MT | 0.0000 | 0.0000 | 0.0000 | 0.0000 | 0.0000 | 0.0000 | 0.0000 | 0.0000 | 0.0000 | 0.0000 | 0.0000 | 1.0000 |
| T14.0763 | neg | MT | 0.0000 | 0.0000 | 0.0000 | 0.0000 | 0.0010 | 0.0000 | 0.0000 | 0.0000 | 0.0002 | 0.0000 | 0.0000 | 0.9988 |
| T14.0764 | neg | MT | 0.0000 | 0.0000 | 0.0001 | 0.0000 | 0.0000 | 0.0003 | 0.0000 | 0.0000 | 0.0002 | 0.0000 | 0.0000 | 0.9994 |
| T14.0765 | neg | MT | 0.0000 | 0.0002 | 0.0002 | 0.0004 | 0.0000 | 0.0334 | 0.0000 | 0.0000 | 0.0002 | 0.0000 | 0.0000 | 0.9656 |
| T14.0766 | neg | MT | 0.0000 | 0.0001 | 0.0000 | 0.0000 | 0.0001 | 0.0000 | 0.0000 | 0.0000 | 0.0000 | 0.0000 | 0.0000 | 0.9998 |
| T14.0767 | neg | MT | 0.0000 | 0.0000 | 0.0005 | 0.0000 | 0.0000 | 0.0019 | 0.0000 | 0.0000 | 0.0000 | 0.0000 | 0.0000 | 0.9976 |
| T14.0769 | neg | MT | 0.0000 | 0.0002 | 0.0000 | 0.0000 | 0.0001 | 0.0002 | 0.0000 | 0.0000 | 0.0000 | 0.0000 | 0.0000 | 0.9995 |
| T14.0770 | neg | MT | 0.0000 | 0.0001 | 0.0000 | 0.0000 | 0.0000 | 0.0000 | 0.0000 | 0.0000 | 0.0000 | 0.0000 | 0.0000 | 0.9999 |
| T14.0773 | neg | MT | 0.0000 | 0.0000 | 0.0000 | 0.0000 | 0.0000 | 0.0000 | 0.0000 | 0.0000 | 0.0000 | 0.0000 | 0.0000 | 1.0000 |
| T14.0774 | neg | MT | 0.0000 | 0.0429 | 0.0000 | 0.0000 | 0.0000 | 0.0000 | 0.0000 | 0.0000 | 0.0001 | 0.0000 | 0.0000 | 0.9570 |
| T14.0775 | R.philipii | MT | 0.0000 | 0.0000 | 0.0000 | 0.0000 | 0.0000 | 0.0000 | 0.0000 | 0.0000 | 0.0000 | 0.0000 | 0.0000 | 1.0000 |
| T14.0092 | R.rhipicephali | MT | 0.0000 | 0.0000 | 0.0000 | 0.0000 | 0.0000 | 0.0000 | 0.0000 | 0.0000 | 0.0000 | 0.0000 | 0.0000 | 1.0000 |
| T14.0730 | R.rhipicephali | MT | 0.0000 | 0.0000 | 0.0000 | 0.0000 | 0.0001 | 0.0000 | 0.0000 | 0.0000 | 0.0000 | 0.0000 | 0.0000 | 0.9999 |
| T14.0762 | R.rhipicephali | MT | 0.0000 | 0.0000 | 0.0000 | 0.0000 | 0.0000 | 0.0000 | 0.0000 | 0.0000 | 0.0000 | 0.0000 | 0.0000 | 1.0000 |
| T14.0768 | R.rhipicephali | MT | 0.0000 | 0.0000 | 0.0000 | 0.0000 | 0.0000 | 0.0000 | 0.0000 | 0.0000 | 0.0001 | 0.0000 | 0.0000 | 0.9999 |
| T14.0408 | neg | PC | 0.0000 | 0.2303 | 0.0000 | 0.0000 | 0.0000 | 0.0000 | 0.0000 | 0.0000 | 0.0000 | 0.0000 | 0.0000 | 0.7697 |
| T14.0409 | neg | PC | 0.0000 | 0.0496 | 0.0000 | 0.0000 | 0.0002 | 0.0007 | 0.0000 | 0.0000 | 0.0001 | 0.0000 | 0.0000 | 0.9494 |
| T14.0410 | neg | PC | 0.0000 | 0.1263 | 0.0000 | 0.0000 | 0.0000 | 0.0001 | 0.0000 | 0.0000 | 0.0000 | 0.0000 | 0.0000 | 0.8736 |
| T14.0411 | neg | PC | 0.0000 | 0.3948 | 0.0000 | 0.0000 | 0.0000 | 0.0000 | 0.0001 | 0.0000 | 0.0004 | 0.0000 | 0.0002 | 0.6045 |
| T14.0420 | neg | PC | 0.0000 | 0.0437 | 0.0000 | 0.0000 | 0.0000 | 0.0000 | 0.0000 | 0.0000 | 0.0000 | 0.0000 | 0.0000 | 0.9563 |
| T14.0422 | neg | PC | 0.0000 | 0.0067 | 0.0000 | 0.0000 | 0.0001 | 0.0000 | 0.0000 | 0.0000 | 0.0000 | 0.0000 | 0.0000 | 0.9932 |
| T14.0423 | neg | PC | 0.0000 | 0.1167 | 0.0000 | 0.0000 | 0.0001 | 0.0000 | 0.0000 | 0.0000 | 0.0000 | 0.0000 | 0.0000 | 0.8832 |
| T14.0434 | neg | PC | 0.0000 | 0.0922 | 0.0000 | 0.0000 | 0.0000 | 0.0000 | 0.0000 | 0.0000 | 0.0001 | 0.0000 | 0.0001 | 0.9076 |
| T14.0436 | neg | PC | 0.0000 | 0.0166 | 0.0000 | 0.0000 | 0.0000 | 0.0000 | 0.0000 | 0.0000 | 0.0000 | 0.0000 | 0.0001 | 0.9833 |
| T14.0399 | R.rhipicephali | PC | 0.0000 | 0.0001 | 0.0000 | 0.0000 | 0.0000 | 0.0000 | 0.0000 | 0.0000 | 0.0001 | 0.0000 | 0.0000 | 0.9997 |
| T14.0404 | R.rhipicephali | PC | 0.0000 | 0.0104 | 0.0001 | 0.0000 | 0.0001 | 0.0000 | 0.0000 | 0.0001 | 0.0000 | 0.0000 | 0.0000 | 0.9893 |
| T14.0407 | R.rhipicephali | PC | 0.0000 | 0.0156 | 0.0000 | 0.0000 | 0.0000 | 0.0000 | 0.0000 | 0.0000 | 0.0000 | 0.0000 | 0.0001 | 0.9843 |
| T14.0418 | R.rhipicephali | PC | 0.0000 | 0.0000 | 0.0000 | 0.0000 | 0.0001 | 0.0000 | 0.0000 | 0.0000 | 0.0000 | 0.0000 | 0.0000 | 0.9999 |
| T14.0419 | R.rhipicephali | PC | 0.0000 | 0.0306 | 0.0000 | 0.0000 | 0.0000 | 0.0000 | 0.0000 | 0.0000 | 0.0000 | 0.0000 | 0.0001 | 0.9693 |
| T14.0428 | R.rhipicephali | PC | 0.0000 | 0.0001 | 0.0000 | 0.0000 | 0.0000 | 0.0000 | 0.0000 | 0.0000 | 0.0000 | 0.0000 | 0.0000 | 0.9999 |
| T14.0433 | R.rhipicephali | PC | 0.0000 | 0.1227 | 0.0000 | 0.0000 | 0.0001 | 0.0000 | 0.0000 | 0.0000 | 0.0000 | 0.0000 | 0.0000 | 0.8772 |
